# Early Antiretroviral Therapy During Primary HIV-1 Infection Results in a Transient Reduction of the Viral Setpoint upon Treatment Interruption

**DOI:** 10.1371/journal.pone.0027463

**Published:** 2011-11-15

**Authors:** Viktor von Wyl, Sara Gianella, Marek Fischer, Barbara Niederoest, Herbert Kuster, Manuel Battegay, Enos Bernasconi, Matthias Cavassini, Andri Rauch, Bernard Hirschel, Pietro Vernazza, Rainer Weber, Beda Joos, Huldrych F. Günthard, the Swiss HIV Cohort Study (SHCS)

**Affiliations:** 1 Division of Infectious Diseases and Hospital Epidemiology, University Hospital Zürich, University of Zürich, Zürich, Switzerland; 2 Research Department of Infection and Population Health, University College London, London, United Kingdom; 3 Division of Infectious Diseases, School of Medicine, University of California San Diego, La Jolla, California, United States of America; 4 Division of Infectious Diseases and Hospital Epidemiology, University Hospital Basel, Basel, Switzerland; 5 Division of Infectious Diseases, Regional Hospital Lugano, Lugano, Switzerland; 6 Division of Infectious Diseases and Hospital Epidemiology, University Hospital Lausanne, Lausanne, Switzerland; 7 University Clinic of Infectious Diseases, University Hospital Bern and University of Bern, Bern, Switzerland; 8 Division of Infectious Diseases, Geneva University Hospital, Geneva, Switzerland; 9 Division of Infectious Diseases, Cantonal Hospital St. Gallen, St. Gallen, Switzerland; Mayo Clinic, United States of America

## Abstract

**Background:**

Long-term benefits of combination antiretroviral therapy (cART) initiation during primary HIV-1 infection are debated.

**Methods:**

The evolution of plasma HIV-RNA (432 measurements) and cell-associated HIV-DNA (325 measurements) after cessation of cART (median exposure 18 months) was described for 33 participants from the Zurich Primary HIV Infection Study using linear regression and compared with 545 measurements from 79 untreated controls with clinically diagnosed primary HIV infection, respectively a known date for seroconversion.

**Results:**

On average, early treated individuals were followed for 37 months (median) after cART cessation; controls had 34 months of pre-cART follow-up. HIV-RNA levels one year after cART interruption were −0.8 log_10_ copies/mL [95% confidence interval −1.2;−0.4] lower in early treated patients compared with controls, but this difference was no longer statistically significant by year three of follow-up (−0.3 [−0.9; 0.3]). Mean HIV-DNA levels rebounded from 2 log_10_ copies [1.8; 2.3] on cART to a stable plateau of 2.7 log_10_ copies [2.5; 3.0] attained 1 year after therapy stop, which was not significantly different from cross-sectional measurements of 9 untreated members of the control group (2.8 log_10_ copies [2.5; 3.1]).

**Conclusions:**

The rebound dynamics of viral markers after therapy cessation suggest that early cART may indeed limit reservoir size of latently infected cells, but that much of the initial benefits are only transient. Owing to the non-randomized study design the observed treatment effects must be interpreted with caution.

## Introduction

The benefits of early initiation of combination antiretroviral treatment (cART) during primary HIV-1 infection (PHI) (henceforth “early cART”) are controversial, because results from previous studies do not seem to be consistent [1], [2], [3], [4]. While some authors reported clinical benefits of early cART in terms of higher CD4 cell counts relative to controls [5], [6], these findings could not be confirmed by others [7], [8], [9]. Also, discrepant reports exist on the impact of early cART on post-treatment viral setpoints, with some studies corroborating [5], [6], [10], [11], [12], [13] and others rejecting the hypothesis of a possible sustained decrease in viral setpoint following treatment with cART during PHI [14], [15], [16], [17], [18]. These studies are difficult to compare however, and there are multiple explanations for the discrepancies, which include differences in timing of cART initiation, type and duration of initial cART, as well as in the choice of controls and the length of follow up [19]. Emerging randomized clinical trials also point toward a beneficial effect of early therapy during acute infection [20], [21].

Moreover, while short to intermediate impacts of early therapy on viral markers are relatively well studied, there are comparably few data on the long term evolution of HIV-RNA and HIV-DNA after cessation of early cART [5], [14]. Previously we have reported differences in HIV-RNA and HIV-DNA setpoints measured after cessation of initial cART between patients diagnosed with PHI who have either started therapy within 60 days of infection (early starters) or after 60 days (later starters) [22]. This analysis included HIV-RNA values measured up to one year after therapy cessation. Here, we aim to describe the long-term dynamics and the correlation of HIV-RNA and -DNA markers before, during and after treatment started at time of PHI.

## Methods

### Ethical Statement

The Zurich Primary HIV Infection Study (ZPHI) has obtained written informed consents from their patients, and the study protocol was approved by the ethics committee of the University Hospital of Zurich. The Swiss HIV Cohort Study (SHCS) has obtained written informed consent from its participants and has been approved by ethical committees of all participating institutions, which are the Kantonale Ethikkommission (KEK) Bern, Bern; the Ethikkommission beider Basel (EKBB), Basel; the comité d'éthique du département de médecine, Hôpitaux Universitaires de Genève, Geneva; the commission d'éthique de la recherche clinique, Faculté de Biologie et de Médecine, Université the Lausanne, Lausanne; the Comitato etico cantonale, Republica e Cantone Ticino, Bellinzona; the Ethikkommission des Kantons St. Gallen, St. Gallen; and the Kantonale Ethikkommission (KEK), Gesundheitsdirektion Kanton Zürich, Zurich.

### Patients

**Figure 1:**
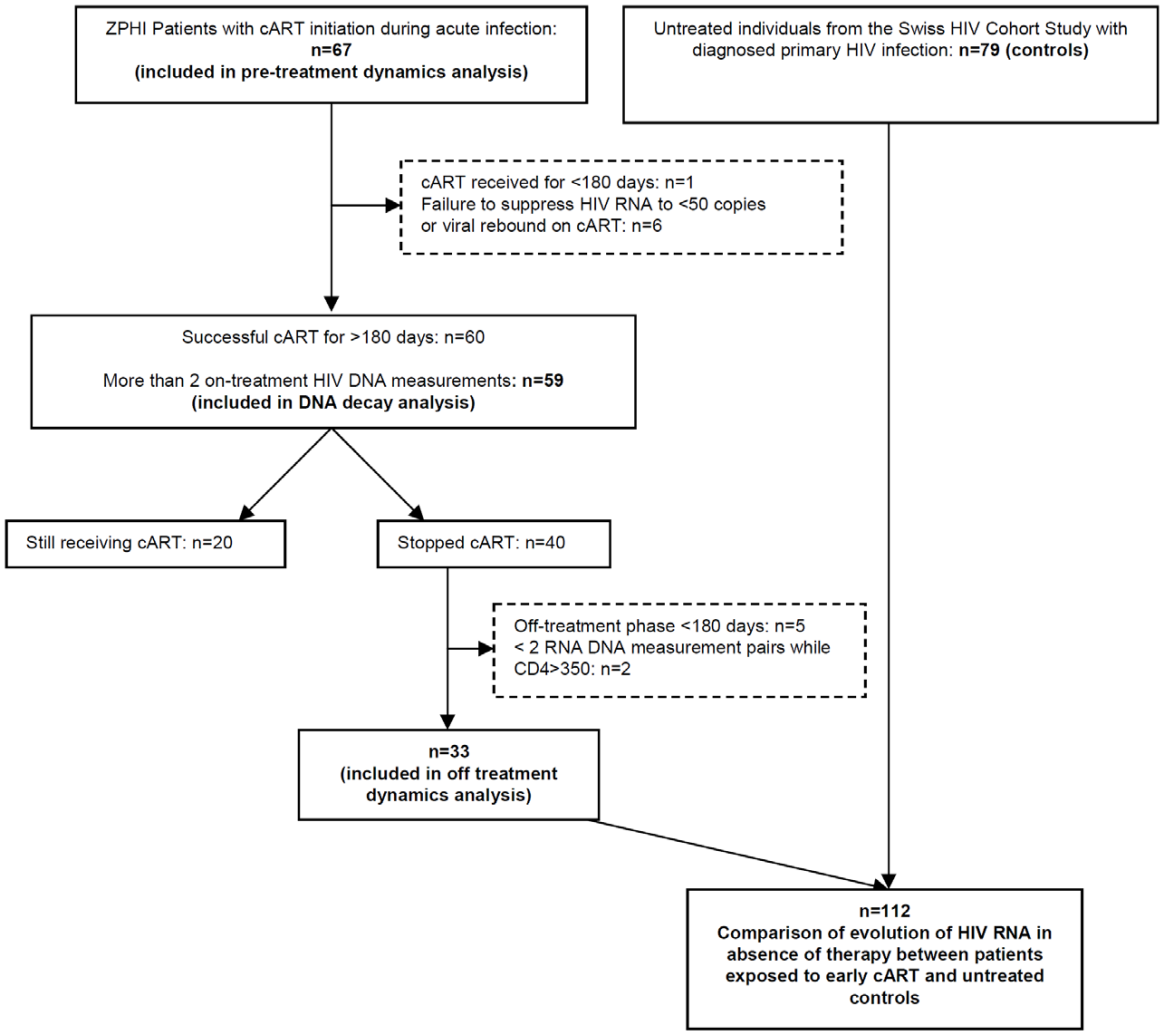
The algorithm used to determine the inclusion (in boxes with continuous bounds) and exclusion (in boxes with interrupted bounds) in pre-defined sub-analyses to explore the effect of early started antiretroviral therapy in primary HIV-infected individuals.

Patients presenting with acute or recent HIV-1 infection were enrolled in the Zurich Primary HIV Study (www.clinicaltrials.gov, NCD00537966) [23], [24] between November 2002 and July 2007. This study is described in details elsewhere [22], [24]. In brief, acutely and recently HIV-1 infected individuals are offered standard first line cART [25], and after one year of viral suppression below detection limits they can elect to stop therapy. Further inclusion criteria for this analysis are outlined in Figure 1. The control group of untreated, chronically infected patients was selected from the Swiss HIV Cohort Study (described in [26]) and consisted of patients with a positive HIV test, clinically diagnosed primary HIV infection and at least 2 HIV-RNA measurements 90 days after the first positive HIV test (to ensure that these patients were no longer in the acute phase). Of those, approximately 41% also had negative and positive HIV-tests performed within 180 days available. A full description of the estimation procedures for the infection date of early treated individuals is given in [22].

For the untreated controls, the infection was assumed to have occurred 30 days prior to the diagnosis of the primary HIV infection.

Although formally two independent studies, all ZPHI participants included in this analysis were also enrolled in the SHCS, and clinical data and laboratory measurements are exchanged anonymously between studies. The ZPHI exclusively recruits participants from the Zurich region, and controls were selected from the remaining SHCS study centers, where early antiretroviral therapy during primary HIV infection was not standard of care at time of study enrolment. Our study design therefore resembles that of a multi-centric study, in which one center exclusively receives the intervention and all other centers recruit controls. Noteworthy, the SHCS is highly representative for the HIV-1 epidemic in Switzerland and encompasses approximately 50% of all HIV-infected, 75% of all individuals receiving antiretroviral therapy, and 75% of all individuals with a CDC stage C diagnosis [27].

The methods for collection of HIV-DNA data are described elsewhere [22], [28], [29]. HIV-RNA in plasma was measured using Amplicor HIV-Monitor version 1.5 (Roche Diagnostics, Basel, Switzerland).

### Statistical methods

Pre-treatment dynamics of HIV-RNA and -DNA were evaluated with linear regression models including linear and quadratic terms (to account for possible non-linear dynamics). Patients only contributed one data point per analysis. The final model was selected based on the basis of adjusted R^2^ values.

The on-treatment dynamics of HIV-RNA was only analyzed descriptively, because it has been extensively studied before [30] and the HIV-RNA assay detection limit of 50 copies/mL precludes sensible modeling analyses of long term decays on therapy. Decay rates of HIV-DNA during treatment were modeled as a two phase decay according to the following equation: 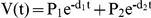, where *d_1_* and *d_2_* are the first- and second-phase decay rates and *P_1_* and *P_2_* are intercepts. To account for the fact that patients contributed several measurements to the analysis we used a non-linear mixed model with random slope and random intercept.

Longitudinal analyses of the evolution of HIV-RNA and -DNA markers after therapy cessation included several measurements per patient and were therefore performed by using linear mixed models with random slope and random intercept. All measurements taken between 30 days after cART stop and 18 months (corresponding to the median follow-up time for HIV-DNA measurements) were used in this analysis. Time since therapy cessation was modeled linearly, with different linear slopes for early starters and untreated controls, or as restricted cubic splines with three knots. The final model was selected by comparison of the Akaike information criterion.

In addition, long term evolution of off-therapy HIV-RNA measurements were compared between patients with early cART initiation and untreated control patients from the SHCS. All measurements taken 30 days after therapy cessation until therapy resumption (early starters) or 90 days after the first positive HIV test until therapy initiation (control group) were considered in the analysis. Data were analyzed with linear mixed models including time as a restricted cubic spline with three knots. Because the magnitude of HIV-RNA is linked with the speed at which patients will start therapy through CD4 cell decline [31], this can introduce a bias into the analysis [19]. For example, individuals with high viral setpoints may have a steeper CD4 decline and may tend to resume therapy earlier, thus lowering the average population viral setpoint. We dealt with this problem by artificially censoring our analysis after 36 months of follow-up, which corresponds to the median post-treatment follow-up time, and, in addition, by using joint mixed models on the full data set, which adjusts longitudinal analyses of viral markers for factors that are associated with the time until therapy is resumed or initiated, such as CD4 cell counts [32].

Statistical analyses were performed using R 2.10 (www.r-project.org) with the NLME library (version 3.1–89) and the JM package (version 0.6.1) [32], and Stata 11.1/SE (Stata Corp. College Station, TX). All analyses were performed on log_10_ transformed data. Model assumptions were checked by plotting fitted values versus residuals and residuals against time. The level of significance was set at p<0.05, and all p-values were two-sided.

## Results

### Patient population

HIV-DNA was measured from 67 patients who started cART within the first four months of their HIV infection. Of these, 59 were finally included in the HIV-DNA decay study and 33 in the cART interruption analysis, respectively (Figure 1). In the full data set of 67 patients the majority of individuals were males who had acquired HIV through homosexual contacts and were infected with HIV subtype B (n =  44, 66%). A further subgroup of notable size (n = 14, 21%) consisted of individuals with heterosexual mode of HIV acquisition and infection with HIV subtypes other than B (predominantly CRF_01, n = 8). All patients initiated cART with protease inhibitors (65 lopinavir, 2 unboosted nelfinavir) and two nucleoside reverse transcriptase inhibitors. Median [interquartile range] baseline CD4 counts and HIV-RNA measurements were 454 cells/µL [407; 500] and 5.3 log_10_ copies/mL [5.1; 5.5], respectively. Median time from infection to the first laboratory measurements (CD4, HIV-RNA, HIV-DNA) and cART initiation was 1.6 months [1.4; 1.8]. The median treatment duration for those individuals who stopped therapy was 18.2 months [15.6; 20.5].

### Dynamics of HIV-DNA and HIV-RNA before early cART initiation

**Figure 2:**
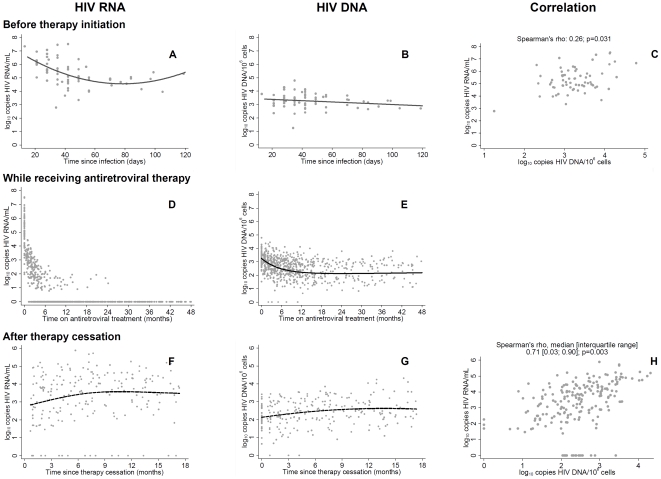
Time courses of and correlations between HIV-DNA and HIV-RNA measurements. Figure 2 depicts time courses of and correlations between HIV-DNA and HIV-RNA measurements obtained before (panels A, B, C), during (panels D, E) and after early antiretroviral therapy initiation (panels F, G, H) in primary HIV-infected individuals. Panels A, B and C show the time dependency of pre-treatment HIV-DNA and -RNA measurements (n = 67 patients). The curves show fits from linear regression including linear and quadratic terms. Final model selection was based on adjusted R^2^ values. Panel A plots plasma HIV-RNA (log_10_ copies/mL) against time since infection, panel B shows Cell associated HIV-DNA (log_10_ copies/million cells), and panel C displays the correlation analysis of pre-treatment HIV-RNA and HIV-DNA. Panels D and E depict the dynamics of HIV-RNA (n =  700 measurements) and HIV-DNA (n = 737 measurements) during treatment with combination antiretroviral therapy in 59 patients. Panel D shows longitudinal HIV-RNA measurements during treatment. Note that undetectable HIV-RNA measurements were artificially set to 0. Panel E shows results from non-linear modeling analyses of the longitudinal course of HIV-DNA under antiretroviral therapy. Panels F and G show the evolution of viral marker pairs (HIV-RNA and HIV-DNA; n = 203 measurements) up to 18 months after therapy cessation (n = 33 patients). Curves were fitted with linear mixed models with random intercepts and random slopes. Panel H displays the correlation between post-therapy HIV-RNA and HIV-DNA measurements. The correlation coefficient shown is the median of 33 individual Spearman correlation analyses for each patient.

As illustrated by Figure 2A, the majority of baseline measurements for the 67 patients were taken early after infection at a time when HIV viral loads are typically very high in most patients. This dynamic appeared to be non-linear in the case of HIV-RNA, because a regression with a linear slope and a non-linear component (i.e. the square of time since infection) showed a better fit to the data (R^2^ = 0.25, F-test p<0.001) than a model with a linear component only (R^2^ = 0.16, F-test p<0.001). In contrast, the decrease of log_10_ HIV-DNA over time was better described by a simple regression with a linear decay (R^2^ = 0.02, F-test p<0.13 vs. R^2^ = 0.01, F-test p<0.26 when including a non-linear component, Figure 2B). Despite the distinct dynamics of HIV-RNA and HIV-DNA, we observed a moderate correlation between these two markers, as shown in Figure 2, panel C.

### Dynamics of HIV-DNA and HIV-RNA on therapy

The dynamics of HIV-DNA and -RNA on therapy was evaluated for the 59 patients with successful cART for at least 180 days. As expected, HIV-RNA dropped rapidly to undetectable levels during treatment (Figure 2D). In contrast, the decay of HIV-DNA occurred much slower. The non-linear modeling analysis shows a two-phase exponential decay (Figure 2E), which had a better model fit than simple one phase decay models. This analysis indicated a fast initial decay of HIV-DNA of 1.22 log_10_ [95% confidence interval 1.04; 1.40] with a half-life of 116 days [93; 153]. The second phase decay was much slower, and decay rates were no longer significantly different from zero (NLMM: −5×10^−5^ [−2×10^−4^; 6×10^−5^]). Thus, HIV-DNA levels remained stable around 2.04 logs_10_ DNA [1.81; 2.27] after approximately 12 months of therapy (Figure 2E).

Interestingly, one individual had several consecutive undetectable HIV-DNA levels during therapy. This male person acquired HIV through heterosexual contacts, was infected with subtype CRF01_AE and had very low baseline HIV-DNA (18 copies/10^6^ cells) and HIV-RNA (609 copies/mL), measured 34 days after HIV infection. HIV-DNA and HIV-RNA measurements became detectable again after therapy cessation (10 copies/10^6^ cells and 100 copies/mL, respectively). However, approximately one year after treatment cessation this individual's HIV-RNA reached 3000 copies/mL and attained 18000 copies/mL after two years.

### Correlation of HIV-RNA and HIV-DNA up to 18 months after cessation of antiretroviral therapy

Next we studied the rebound of HIV-RNA and -DNA after cessation of cART and the correlation of these two viral markers. For this analysis, all measurements taken 30 days after therapy stop and up to 18 months later were considered. The best curve fit for HIV-RNA was a cubic spline with knots placed at 1.2, 6.4, and 14.4 months, yielding a curve with a steep initial increase and a relatively flat region after 9 months (Figure 2F). The best-fit curve for HIV-DNA, based on a linear and a quadratic term, looked very similar to the dynamics in HIV-RNA (Figure 2G). Consequently, the correlation of HIV-RNA and HIV-DNA measurements was quite strong in the post-therapy phase (Figure 2H). When performing separate Spearman correlation analyses for each patient, the median Spearman correlation coefficient over all individuals was 0.71, which was significantly different from 0 by the Wilcoxon signed rank test (p = 0.003).

We further aimed to find predictors for post-treatment HIV-RNA levels. We hypothesized that the following parameters may possibly have an impact: duration of antiretroviral therapy, timing of therapy initiation (≤60 after infection or later, as described in [22]), and pre-treatment levels of HIV-DNA and CD4 cell counts. Analyses were further adjusted for patient sex, age, ethnicity, and mode of HIV-acquisition. As previously reported [22] and shown in Table 1, the only parameter of interest showing statistical significance in the multivariable model was timing of treatment: individuals with antiretroviral therapy initiation ≤60 days after infection (n = 24) tended to have lower post-treatment HIV-RNA levels than patients with therapy start between 61 and 120 days (n = 9). The appropriateness of the 60 day break point for this analysis was further confirmed by use of fractional polynomial regression (not shown) [33].

**Table 1:** Predictors for post-therapy HIV-RNA levels collected up to 18 months after stop of antiretroviral therapy (included 203 HIV-RNA measurements from 33 patients). Time was modeled as a restricted cubic spline with three knots (coefficients not shown).

	Univariable	P-value	Multivariable	P-value
	regression coefficient		regression coefficient	
	[95% CI]		[95% CI]	
Male sex	0.29 [−0.99 to 1.57]	0.656	−0.90 [−2.27 to 0.48]	0.202
White ethnicity (vs. non-white)	−0.79 [−2.59 to 1.00]	0.386	−0.25 [−1.86 to 1.37]	0.766
Age (per 10 years older)	−0.21 [−0.67 to 0.26]	0.381	0.41 [−0.11 to 0.94]	0.124
Acquired HIV through homosexual contacts (vs. heterosexual contacts)	0.94 [0.12 to 1.76]	0.025	1.37 [0.32 to 2.42]	0.01
CD4 cell count before therapy start (per 10 cells higher)	−0.02 [−0.04 to 0.01]	0.163	−0.02 [−0.04 to 0.01]	0.137
Log_10_ HIV-DNA before therapy start	0.45 [−0.24 to 1.14]	0.204		
Duration of initial treatment with combination antiretroviral therapy (per month longer)	0.02 [−0.05 to 0.08]	0.614		
Treatment initiation <60 days after HIV infection (vs. later)	−0.84 [−1.78 to 0.11]	0.082	−1.01 [−1.98 to −0.04]	0.041

### Long term evolution of viral markers

**Figure 3:**
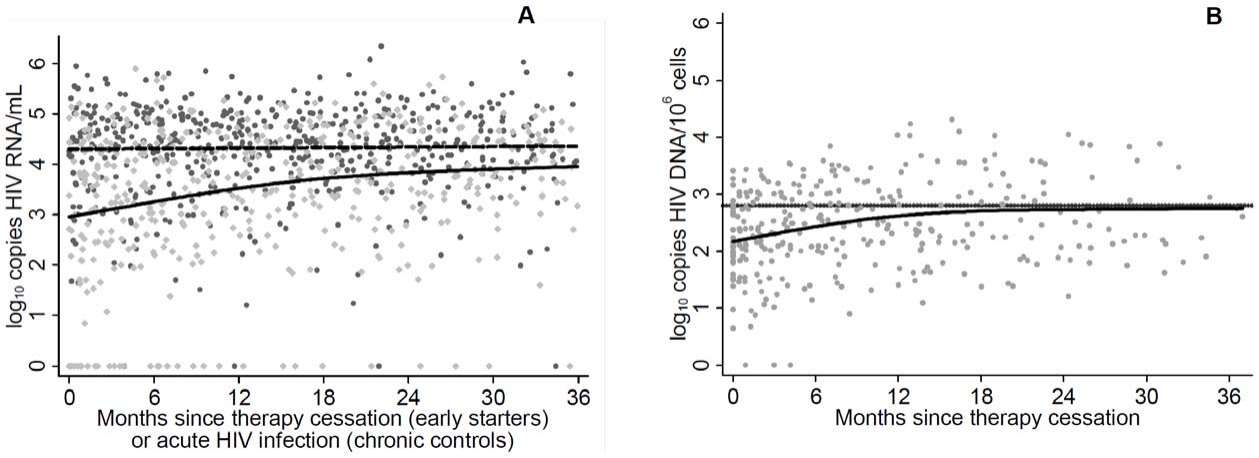
Long-term evolution of viral markers. Panel A displays the evolution of HIV-RNA measurements up to 36 months after therapy cessation in 33 individuals with therapy initiation during PHI (n = 432 measurements, light grey symbols) and 79 chronically infected participants of the Swiss HIV Cohort Study (n = 545 measurements, dark symbols). Panel B shows longitudinal HIV-DNA measured in early starters (n = 325 measurements). Note that the dotted line represents the average of 9 randomly selected cross-sectional measurements from control patients.

We also studied the long term evolution of HIV-RNA upon cART cessation among patients with early cART initiation and the untreated controls. Their characteristics are compared in Table 2. Relevant differences were observed in the magnitude of baseline CD4 with a 160 cells higher median value in the control group and, as expected by design, in the frequency and timing of therapy initiation. As shown in Figure 3A, HIV-RNA levels in early cART initiators were generally below those from the control group. Predictions from the best-fit model adjusted for age, sex, ethnicity and mode of HIV acquisition indicate that after 1 year of follow-up early starters on average had -0.74 log_10_ copies lower HIV-RNA levels/mL ([95% confidence interval −1.17; −0.31], p<0.001). This difference diminished over time, however, and after 2 and 3 years was reduced to −0.42 ([−0.91; 0.74], p = 0.096) and −0.25 ([−.0.94; 0.43], p = 0.468) log_10_ copies HIV-RNA/mL, respectively. While, overall, there was no difference in rates of treatment initiation (controls, median time from first positive HIV test +90 days to therapy start: 30 months) or resumption (early treated individuals, median time after stop of first therapy: 37 months, log rank p = 0.64), Cox proportional hazard regression suggested an association of CD4 count levels at baseline, which differed between early treated individuals and controls, with the probability for therapy initiation (Table S1). Nevertheless an extension of follow-up time to 48 months in combination with a joint modeling approach of HIV-RNA evolution and time to therapy initiation yielded almost identical results (Table S2). Additional sensitivity analyses were performed by including all control patients (n = 79) and only early treated individuals with a therapy start within 60 days after infection (n = 24), which was associated with lower viral load levels up to 18 months after therapy cessation in the analysis shown in Table 1, or by restricting the control population to individuals who, in addition to a clinical primary HIV diagnosis, also had negative and positive HIV tests not longer than 180 days apart (n = 32). Neither of these sensitivity analyses altered our conclusions, because the shapes of HIV-RNA trajectories did not change substantially (Table S2).

**Table 2:** Comparison of baseline characteristics for early treated individuals (n = 33) and untreated controls with a clinical diagnosis of primary HIV infection (n = 79).

	Early treated individuals	Controls	P-value
N	33 (100.0)	79 (100.0)	
Age	39 [36]; [42]	37 [35]; [39]	0.311
Baseline CD4 count	499 [436; 561]	661 [606; 717]	<0.001
Baseline HIV-RNA	4.9 [4.5; 5.2]	4.9 [4.6; 5.1]	0.990
Ethnicity			0.704
Black	0 (0.0)	1 (1.3)	
Other	2 (6.1)	2 (2.5)	
White	31 (93.9)	76 (96.2)	
Year of HIV-1 Infection	2004 [2003; 2004]	2005 [2004; 2005]	0.044
Clinical diagnosis of primary HIV infection	32 (97.0)	79 (100.0)	0.295
Mode of HIV-1 acquisition			0.327
Heterosexual	10 (30.3)	22 (27.8)	
Homosexual	22 (66.7)	47 (59.5)	
Other	1 (3.0)	10 (12.7)	
Male sex	29 (87.9)	66 (83.5)	0.774
HIV Subtype			<0.001
CRF 01_AE	8 (24.2)	1 (1.3)	
CRF 02_AG	0 (0.0)	5 (6.3)	
B	22 (66.7)	55 (69.6)	
Other	3 (9.1)	18 (22.8)	
Time from infection to baseline (months)	20 [17]; [23]	6 [5]; [8]	<0.001
Ever started antiretroviral therapy	33 (100)	51 (64.6)	<0.001
Time to therapy initiation (months)	1.7 [1.5; 1.9]	40.5 [34.3; 46.6]	<0.001

The baseline for this analysis was set at 30 days after therapy cessation for the intervention group and at 90 days after the first positive HIV test for the control group.

The dynamics of HIV-DNA over 36 months after therapy cessation resembled those of HIV-RNA by showing an increase in the initial phase from approximately 2 log_10_ copies/10^6^ cells (Figure 3B) after treatment stop to 2.62 log_10_ copies/10^6^ cells [95% confidence interval 2.36; 2.88] and remaining at a stable level of approximately 2.7 log_10_ copies HIV-DNA/10^6^ cells thereafter (estimate for month 24: 2.73 [2.45; 3.02]; month 36: 2.75 [2.35; 3.15]). Of note, this plateau was similar to the average of 9 randomly selected measurements of HIV-DNA from the untreated control group (2.8 log_10_ copies HIV-DNA, [2.48; 3.11], indicated by dotted line in Figure 3B). The joint modeling analysis yielded a similar longitudinal post-therapy course for HIV-DNA in early treated individuals (not shown).

## Discussion

In this analysis we studied the evolution of HIV-RNA and HIV-DNA measurements before (n = 67), during (n = 59) and after antiretroviral therapy (n = 33) in individuals with therapy initiation within 4 months after HIV infection. As our assessment of HIV-DNA measurements sampled before therapy initiation has shown, DNA levels declined during the first few weeks after infection, although this decrease was far less pronounced than for HIV-RNA. Moreover, only a moderate correlation between pre-treatment HIV-RNA and HIV-DNA was observed, which contrasts starkly with the strong correlation of these parameters after therapy cessation. This suggests that, at the time of pre-treatment data collection, the steady state of virus production and decay was not yet established. Our observation that pre-treatment HIV-DNA values were not predictive for post-treatment HIV-RNA levels is consistent with this hypothesis.

While HIV-RNA measurements rapidly attained undetectable levels after treatment initiation, HIV-DNA levels were much slower to respond and, with the exception of one patient (Figure 3B), never reached undetectable levels. On average a level of 2 log_10_ copies HIV-DNA persisted despite a median exposure to combination therapy with boosted protease inhibitors of 18 months, and we observed no evidence that a further extension of therapy duration may yield any benefit in terms of HIV-DNA reduction. This is not surprising given that the PBMC associated DNA levels we measured during the treatment phase largely reflect proviral DNA that is integrated into the host DNA and exhibit a very long decay half life [34], [35]. On a broader view, this finding confirms conventional wisdom that latent HIV-DNA reservoirs cannot be purged by currently available antiretroviral therapies even when started very early [36].

After cessation of antiretroviral therapy, there was a slow increase in HIV-DNA, which seemed to level off after approximately 1 year. In contrast, there was an almost instantaneous rebound from undetectable to detectable HIV-RNA and a continuous rise in levels over the first 18 months after treatment stop. However, when including follow-up data for up to 36 months there was also evidence for a deceleration of increase rates for HIV-RNA levels, similar to the dynamics of HIV-DNA. Indeed, our statistical models suggest that, three years post therapy, individuals who had undergone early treatment almost attained the same HIV-RNA levels as untreated controls, whereas in the first year after treatment stop HIV-RNA levels were still notably different between early cART starters and untreated controls. Similar rebound dynamics of post-treatment HIV-RNA have previously been reported by other studies [5], [14], [18].

The present study offers possible explanations for the differing impact assessments of early therapy on post-treatment viral loads. As indicated by our 18 month analysis (Table 1 and [22]) and data from other studies [6], [11] the timing of treatment initiation may be of importance, with very early treatment initiation generally yielding better outcomes in terms of lower HIV-RNA levels. Furthermore, discrepant results may have arisen from differences in post-therapy follow-up time for outcome assessment, with shorter analysis time frames between 24 and 48 weeks after therapy cessation more likely pointing towards a benefit of early therapy [12], [22], [37], and studies following the HIV-RNA trajectories after therapy stop generally finding decreasing differences in viral loads between early treated individuals and controls [5], [6], [15], [18]. The present analysis adds to these longitudinal observations by specifically addressing statistical problems of non-random censoring of follow-up, meaning that patients with faster HIV-1 progression and potentially higher HIV-RNA loads initiate therapy earlier, and confounding by severity of primary HIV-1 infection symptoms [19], [37].

Some limitations of this study should be noted, most importantly that our analysis is observational and therefore treatment strategies were not subject to randomization. Our findings must therefore be interpreted with caution. Systematic differences between the group of early treated individuals and untreated controls may have affected our analysis, such as the severity of the acute retroviral syndrome, which has been correlated with the magnitude of setpoint viral load. If heavily symptomatic primary infected individuals were more likely to receive early therapy, this could possibly increase the group average of post-therapy viral loads of early treated individuals, therefore diminishing the true treatment effect. A second limitation concerns differences regarding the time axes for early treated individuals and controls. By design, the early treated individuals will have been infected for a longer period of time (20 months) than controls (6 months) in the treatment effects analysis, because early starters will in minimum have received 6 months of therapy, whereas the baseline in untreated controls was set at 90 days after the first positive HIV test. While this systematic difference could have confounded our analysis, for instance via a time dependency of immune response or co-receptor switch, a large effect on the observed treatment effects seems implausible. Additionally, diagnoses of primary HIV infections are not based on a standardized protocol within the SHCS, and thus infection date estimates from untreated controls are less precise than those from early treated individuals. Restricting this imprecision to 3 months by only including controls with negative and positive HIV tests within 6 months (n = 32) in a sensitivity analysis had little impact on our estimates of treatment effects, however (Table S2).

Taken together, these data suggest that the initiation of antiretroviral therapy during PHI may lead to reductions of post-therapy HIV-RNA and HIV-DNA levels, but further demonstrate that these initial benefits may be waning over time. The major mechanism behind our finding is most likely the limited size of the long-lived latent reservoir [38], [39], [40] by an abrogated establishment of this reservoir by early cART, as has been shown by viral outgrowth assays [26], by profound depletion of transcriptionally active cells [29] and by reduced amounts of proviral DNA in PBMC [22]. The post-treatment dynamics of viral markers uncovered in the current analysis are indeed suggestive for such an effect of early therapy. However, these analyses also show that the establishment of reservoirs is merely postponed: After approximately 12 and 36 months, HIV-DNA and HIV-RNA levels resembled those from untreated chronic control patients. Given the transient nature of effects on viral markers, the current lack of ways to exploit these reductions in HIV-RNA and HIV-DNA for patient benefit, and against the background of recent findings of detrimental effects of viral replication on morbidity and mortality [41], [42] these data suggest that therapy resumption in early treated individuals should not be deferred for too long [25].

## Supporting Information

Table S1 **Factors associated with treatment resumption (early treated individuals, n = 33) or treatment initiation (controls, n = 79) in univariable and multivariable Cox regression models.**
(DOC)

Table S2 **Estimates of HIV-RNA levels at 12, 24, and 36 months after baseline from linear mixed models and joint models.** Estimates of difference in HIV-RNA between early starters and controls that printed in bold face are statistically significant at the 5% level.
(DOC)

## References

[1] Kinloch-de Loes S (2006). Treatment of acute HIV-1 infection: is it coming of age?. J Infect Dis.

[2] Smith DE, Walker BD, Cooper DA, Rosenberg ES, Kaldor JM (2004). Is antiretroviral treatment of primary HIV infection clinically justified on the basis of current evidence?. AIDS.

[3] Cellerai C, Little SJ, Loes SK (2008). Treatment of acute HIV-1 infection: are we getting there?. Curr Opin HIV AIDS.

[4] Bell SK, Little SJ, Rosenberg ES (2010). Clinical management of acute HIV infection: best practice remains unknown.. J Infect Dis.

[5] Fidler S, Fox J, Touloumi G, Pantazis N, Porter K (2007). Slower CD4 cell decline following cessation of a 3 month course of HAART in primary HIV infection: findings from an observational cohort.. AIDS.

[6] Hecht FM, Wang L, Collier A, Little S, Markowitz M (2006). A multicenter observational study of the potential benefits of initiating combination antiretroviral therapy during acute HIV infection.. J Infect Dis.

[7] Pantazis N, Touloumi G, Vanhems P, Gill J, Bucher HC (2008). The effect of antiretroviral treatment of different durations in primary HIV infection.. AIDS.

[8] Seng R, Goujard C, Desquilbet L, Sinet M, Rouzioux C (2008). Rapid CD4+ Cell Decrease After Transient cART Initiated During Primary HIV Infection (ANRS PRIMO and SEROCO Cohorts).. J Acquir Immune Defic Syndr.

[9] Streeck H, Jessen H, Alter G, Teigen N, Waring MT (2006). Immunological and virological impact of highly active antiretroviral therapy initiated during acute HIV-1 infection.. J Infect Dis.

[10] Rosenberg ES, Altfeld M, Poon SH, Phillips MN, Wilkes BM (2000). Immune control of HIV-1 after early treatment of acute infection.. Nature.

[11] Steingrover R, Pogany K, Fernandez Garcia E, Jurriaans S, Brinkman K (2008). HIV-1 viral rebound dynamics after a single treatment interruption depends on time of initiation of highly active antiretroviral therapy.. AIDS.

[12] Volberding P, Demeter L, Bosch RJ, Aga E, Pettinelli C (2009). Antiretroviral therapy in acute and recent HIV infection: a prospective multicenter stratified trial of intentionally interrupted treatment.. AIDS.

[13] Hocqueloux L, Prazuck T, Avettand-Fenoel V, Lafeuillade A, Cardon B (2010). Long-term immunovirologic control following antiretroviral therapy interruption in patients treated at the time of primary HIV-1 infection.. AIDS.

[14] Desquilbet L, Goujard C, Rouzioux C, Sinet M, Deveau C (2004). Does transient HAART during primary HIV-1 infection lower the virological set-point?. AIDS.

[15] Kaufmann DE, Lichterfeld M, Altfeld M, Addo MM, Johnston MN (2004). Limited durability of viral control following treated acute HIV infection.. PLoS Med.

[16] Markowitz M, Jin X, Hurley A, Simon V, Ramratnam B (2002). Discontinuation of antiretroviral therapy commenced early during the course of human immunodeficiency virus type 1 infection, with or without adjunctive vaccination.. J Infect Dis.

[17] Hoen B, Fournier I, Lacabaratz C, Burgard M, Charreau I (2005). Structured treatment interruptions in primary HIV-1 infection: the ANRS 100 PRIMSTOP trial.. J Acquir Immune Defic Syndr.

[18] Steingrover R, Garcia EF, van Valkengoed IG, Bekker V, Bezemer D (2010). Transient lowering of the viral set point after temporary antiretroviral therapy of primary HIV type 1 infection.. AIDS Res Hum Retroviruses.

[19] Clements M, Law M, Pedersen C, Kaldor J (2003). Estimating the effect of antiretroviral treatment during HIV seroconversion: impact of confounding in observational data.. HIV Med.

[20] Fidler S, SPARTAC Investigators (2011). The effect of short-course antiretroviral therapy in primary HIV infection: final results from an international randomised controlled trial; SPARTAC..

[21] Hogan C, DeGruttola V, Daar E, Sun X, Del Rio C (2010). A Finite Course of ART during Early HIV-1 Infection Modestly Delays Need for Subsequent ART Initiation: ACTG A5217, the SETPOINT Study..

[22] Gianella S, Von Wyl V, Fischer M, Niederoest B, Battegay M (2011). Effect of early antiretroviral therapy during primary HIV-1 infection on cell-associated HIV-1 DNA and plasma HIV-1 RNA.. Antivir Ther.

[23] Aceto L, Karrer U, Grube C, Oberholzer R, Hasse B (2005). Primary HIV-1 infection in Zurich: 2002–2004.. Praxis (Bern 1994).

[24] Rieder P, Joos B, von Wyl V, Kuster H, Grube C (2010). HIV-1 transmission after cessation of early antiretroviral therapy among men having sex with men.. AIDS.

[25] Thompson MA, Aberg JA, Cahn P, Montaner JS, Rizzardini G (2010). Antiretroviral treatment of adult HIV infection: 2010 recommendations of the International AIDS Society-USA panel.. JAMA.

[26] Ledergerber B, Egger M, Opravil M, Telenti A, Hirschel B (1999). Clinical progression and virological failure on highly active antiretroviral therapy in HIV-1 patients: a prospective cohort study. Swiss HIV Cohort Study.. Lancet.

[27] Schoeni-Affolter F, Ledergerber B, Rickenbach M, Rudin C, Gunthard HF (2010). Cohort profile: the Swiss HIV Cohort study.. Int J Epidemiol.

[28] Althaus CF, Gianella S, Rieder P, von Wyl V, Kouyos RD (2010). Rational design of HIV-1 fluorescent hydrolysis probes considering phylogenetic variation and probe performance.. J Virol Methods.

[29] Schmid A, Gianella S, von Wyl V, Metzner KJ, Scherrer AU (2010). Profound depletion of HIV-1 transcription in patients initiating antiretroviral therapy during acute infection.. PLoS One.

[30] Perelson AS, Neumann AU, Markowitz M, Leonard JM, Ho DD (1996). HIV-1 dynamics in vivo: virion clearance rate, infected cell life-span, and viral generation time.. Science.

[31] Ledergerber B, Lundgren JD, Walker AS, Sabin C, Justice A (2004). Predictors of trend in CD4-positive T-cell count and mortality among HIV-1-infected individuals with virological failure to all three antiretroviral-drug classes.. Lancet.

[32] Rizopoulos D, Verbeke G, Lesaffre E, Vanrenterghem Y (2008). A two-part joint model for the analysis of survival and longitudinal binary data with excess zeros.. Biometrics.

[33] Royston P, Ambler G, Sauerbrei W (1999). The use of fractional polynomials to model continuous risk variables in epidemiology.. Int J Epidemiol.

[34] Strain MC, Little SJ, Daar ES, Havlir DV, Gunthard HF (2005). Effect of treatment, during primary infection, on establishment and clearance of cellular reservoirs of HIV-1.. J Infect Dis.

[35] Koelsch KK, Liu L, Haubrich R, May S, Havlir D (2008). Dynamics of total, linear nonintegrated, and integrated HIV-1 DNA in vivo and in vitro.. J Infect Dis.

[36] Richman DD, Margolis DM, Delaney M, Greene WC, Hazuda D (2009). The challenge of finding a cure for HIV infection.. Science.

[37] Lampe FC, Porter K, Kaldor J, Law M, Kinloch-de Loes S (2007). Effect of transient antiretroviral treatment during acute HIV infection: comparison of the Quest trial results with CASCADE natural history study.. Antivir Ther.

[38] Wong JK, Hezareh M, Gunthard HF, Havlir DV, Ignacio CC (1997). Recovery of replication-competent HIV despite prolonged suppression of plasma viremia.. Science.

[39] Finzi D, Hermankova M, Pierson T, Carruth LM, Buck C (1997). Identification of a reservoir for HIV-1 in patients on highly active antiretroviral therapy.. Science.

[40] Chun TW, Stuyver L, Mizell SB, Ehler LA, Mican JA (1997). Presence of an inducible HIV-1 latent reservoir during highly active antiretroviral therapy.. Proc Natl Acad Sci U S A.

[41] Kuller LH, Tracy R, Belloso W, De Wit S, Drummond F (2008). Inflammatory and coagulation biomarkers and mortality in patients with HIV infection.. PLoS Med.

[42] El-Sadr WM, Lundgren JD, Neaton JD, Gordin F, Abrams D (2006). CD4+ count-guided interruption of antiretroviral treatment.. N Engl J Med.

