# Supplementary material for: Early Antiretroviral Therapy During Primary HIV-1 Infection Results in a Transient Reduction of the Viral Setpoint upon Treatment Interruption
Source: PLoS One. 2011 Nov 15;6(11):e27463. doi: 10.1371/journal.pone.0027463 (PMC3216952; doi:10.1371/journal.pone.0027463)
Supplement: Table S1 — Factors associated with treatment resumption (early treated individuals, n = 33) or treatment initiation (controls, n = 79) in univariable and multivariable Cox regression models. (DOC) [file pone.0027463.s001.doc]

|  | **Univariable Hazard Ratio  [95% Confidence Interval]** | **Multivariable Hazard Ratio  [95% Confidence Interval]** |
| --- | --- | --- |
|  |  |  |
| Belonging to control group | 1.18 [0.71-1.95] | 0.99 [0.56-1.76] |
| Female sex | 0.93 [0.49-1.79] | 0.84 [0.35-1.98] |
| Mode of HIV acquisition |  |  |
| Heterosexual risks | Reference | Reference |
| Homosexual risks | 0.78 [0.47-1.29] | 0.69 [0.35-1.38] |
| Injecting drug use | 0.48 [0.17-1.39] | 0.41 [0.13-1.27] |
| White ethnicity (vs. other) | 1.06 [0.37-3.04] | 0.88 [0.30-2.64] |
| Age groups (by 33 percentiles) |  |  |
| 18-33 | Reference | Reference |
| 34-39 | 1.14 [0.64-2.04] | 1.26 [0.66-2.39] |
| 40-64 | 1.08 [0.62-1.89] | 0.95 [0.51-1.76] |
| CD4 cell count at baseline (by 33 percentiles) |  |  |
| 361-557 | Reference | Reference |
| 564-769 | **0.43 [0.23-0.77]** | **0.39 [0.21-0.74]** |
| 788-1536 | **0.49 [0.28-0.84]** | **0.49 [0.27-0.88]** |
|  |  |  |
| Estimates printed in bold face are statistically significant at the 5% level. | |  |
