# Supplementary material for: Early Antiretroviral Therapy During Primary HIV-1 Infection Results in a Transient Reduction of the Viral Setpoint upon Treatment Interruption
Source: PLoS One. 2011 Nov 15;6(11):e27463. doi: 10.1371/journal.pone.0027463 (PMC3216952; doi:10.1371/journal.pone.0027463)
Supplement: Table S2 — Estimates of HIV-RNA levels at 12, 24, and 36 months after baseline from linear mixed models and joint models. Estimates of difference in HIV-RNA between early starters and controls that printed in bold face are statistically significant at the 5% level. (DOC) [file pone.0027463.s002.doc]

| **Linear Mixed Models** | |  |  |
| --- | --- | --- | --- |
|  |  |  |  |
| **Base Model** |  |  |  |
| Time after baseline | Early Starters (n=33) | Controls (n=79) | Difference in HIV RNA |
| 12 Months | 3.53 [3.13-3.93] | 4.33 [4.15-4.52] | **0.80 [0.37-1.24]** |
| 24 Months | 3.85 [3.47-4.23] | 4.33 [4.16-4.50] | **0.48 [0.06-0.90]** |
| 36 Months | 3.99 [3.51-4.47] | 4.28 [3.98-4.58] | 0.29 [-0.27-0.86] |
|  |  |  |  |
| **Only including early treated individuals with therapy start within 60 days after infection (n=24)** | | | |
| Time after baseline | Early Starters (n=24) | Controls (n=79) | Difference in HIV RNA |
| 12 Months | 3.37 [2.87-3.88] | 4.33 [4.14-4.51] | **0.95 [0.42-1.49]** |
| 24 Months | 3.76 [3.27-4.25] | 4.33 [4.16-4.50] | **0.57 [0.05-1.08]** |
| 36 Months | 3.95 [3.37-4.53] | 4.29 [3.98-4.60] | 0.34 [-0.31-0.99] |
|  |  |  |  |
| **Only including controls with documented seroconversion with negative and positive HIV test within 180 days** | | | |
| Time after baseline | Early Starters (n=33) | Controls (n=32) | Difference in HIV RNA |
| 12 Months | 3.55 [3.14-3.95 | 4.24 [4.00-4.47] | **0.69 [0.22-1.16]** |
| 24 Months | 3.85 [3.46-4.24] | 4.15 [3.96-4.33] | 0.30 [-0.13-0.73] |
| 36 Months | 3.98 [3.49-4.46] | 4.06 [3.64-4.47] | 0.08 [-0.56-0.72] |
|  |  |  |  |
| **Joint Modelling Approach** | |  |  |
|  |  |  |  |
| Time after baseline | Early Starters (n=33) | Controls (n=79) | Difference in HIV RNA |
| 12 Months | 3.46 [3.15-3.76] | 4.31 [4.13-4.50] | **0.85 [0.49-1.23]** |
| 24 Months | 3.94 [3.67-4.22] | 4.31 [4.14-4.48] | **0.36 [0.03-0.70]** |
| 36 Months | 4.35 [4.11-4.60] | 4.35 [4.18-4.53] | 0.00 [-0.31-0.31] |
|  |  |  |  |
